# Modelling the Mechanical Properties of Architected Cellular Solids for Structural Applications: A Review

**DOI:** 10.3390/ma19132711

**Published:** 2026-06-24

**Authors:** Jorge Luis Flores Alarcón, Rafael Schouwenaars, Armando Ortiz, Leopoldo Ruiz-Huerta, Manuel Farid Azamar, Ignacio Alejandro Figueroa

**Affiliations:** 1 Instituto de Investigaciones en Materiales, Universidad Nacional Autónoma de México (UNAM), Ciudad Universitaria, Coyoacán, Mexico City 04510, Mexico; jorge.alarcon@ingenieria.unam.edu; 2 Departamento de Materiales y Manufactura, Facultad de Ingeniería, Edificio O, Universidad Nacional Autónoma de México (UNAM), Ciudad Universitaria, Coyoacán, Mexico City 04510, Mexico; armandoo@unam.mx (A.O.); manuel.azamar.j@comunidad.unam.mx (M.F.A.); 3 Materials Science and Technology, Department of Electromechanical, Systems and Metals Engineering, Ghent University, Technologiepark 46, 9052 Ghent, Belgium; 4 Instituto de Ciencias Aplicadas y Tecnología (ICAT), Universidad Nacional Autónoma de México (UNAM), Circuito Exterior S/N, Ciudad Universitaria, Mexico City 04510, Mexico; leoruiz@unam.mx; 5 National Laboratory for Additive and Digital Manufacturing (MADiT), Universidad Nacional Autónoma de México (UNAM), Circuito Exterior S/N, Ciudad Universitaria, Mexico City 04510, Mexico

**Keywords:** cellular solid, modelling, stiffness, yield locus, trusses, triply periodic minimal surface

## Abstract

Among a broad range of promising applications, the use of cellular solids as lightweight structural components is an important field of research that requires reliable predictions of their stiffness and strength. Predictive and general models should not depend on extensive parameter-fitting experiments and should not rely on computationally intensive numerical calculations for each new set of geometric parameters and loading conditions. An overview of models for 2D, 2.5D, and three-dimensional structures will be presented. Most 2D and 2.5D models neglect out-of-plane behaviour and the face sheets used in sandwich panels. 3D studies, mainly by finite element models (FEMs), are often limited to a narrow set of geometries and simple loading conditions. Elastic anisotropy is well covered, but calculating yield surfaces remains a challenge. Simplified models based on structural mechanics are rare and often limited in scope. They offer a flexible, computationally efficient approach for simulating truss-based materials. For more advanced designs, parameter-based FEMs must be developed for any loading condition to facilitate the generalised incorporation of 3D cellular solids in mechanical design. Artificial intelligence and machine learning are promising approaches for making optimal use of experimental and FEM results across multidimensional parameter spaces.

## 1. Introduction

Cellular solids (CS) are found in nature, where evolution has optimised tissues to achieve low density, high stiffness, and high strength. They exhibit enhanced functionality due to their superior topologies and large surface area. Bird beaks and bones are examples of structures with a solid cover attached to a complex cellular core with density gradients [1]. Wood, cork, sponge, coral, and cancellous bone are other examples of natural cellular materials with a tubular structure supported by an inner core of honeycomb/foam-like cells [2,3].

Humans have utilised these natural cellular materials for centuries. For instance, pyramids in Egypt contain wooden artefacts that are over 5000 years old, and cork was used in wine bottles during Roman times [4]. The oldest bone tools have been reported to be approximately 1.5 million years old [5], while the oldest reported structural use of wood dates back to 476,000 years [6]. Bamboo is one of the most important building materials globally [7] and presents an impressive hierarchical cellular structure that directly determines its mechanical properties [8,9].

Motivated by the advantages of natural cellular materials, the materials science community has focused on developing advanced lightweight cellular structures. An early and authoritative review of the development of metallic foams was provided by Banhart [10]. More recent reviews were provided by Atwater et al. [11] and Wan et al. [12]. Foams are a form of cellular structure that are manufactured by injecting gas, mixing a foaming agent into molten metal, or through spontaneous gas-forming reactions, as illustrated in ceramic foams for structural and chemical applications [13,14]. Metallic foams are highly reliable and robust for industrial and commercial applications, such as energy absorption [15], lightweighting [16], thermal management, vibration damping, acoustic dissipation [17], and as carriers for catalysis [18], but precise control over cell morphology and process-related defects can pose challenges.

Cellular materials with regular structures are found in honeycombs and lattices. Early reviews in the field were provided by Evans et al. [19], who focused on estimating mechanical and thermal properties, and by Fleck et al., who provided a timeline of their development and case studies predicting their properties [20]. An important topic in these works is the use of honeycombs and lattices in sandwich panels for aerospace applications. Benedetti et al. [21] reviewed architected materials, with a focus on fatigue properties. A contemporary review by Du Plessis et al. [22] describes the considerable range of cellular materials produced by additive manufacturing.

Architectured materials have gained popularity for high-performance components in aerospace, biomedical, energy, sports, and automotive applications. The replacement of internal solid volumes with lattice structures, which offer comparable strength in the aerospace industry [23], requires the development of architected materials. Automotive applications include prototyping, rapid fabrication, and repair of industrial tools, such as dies and punches [24]. Multiple parts that previously required assembly are now built from architected materials [22,25]. Architected cellular structures are also of great interest in the biomedical field. Ti-based cellular biomaterials were reviewed by Tyagi et al. [26]. Prostheses with porous structures show reduced stress and improved osseointegration [27].

The core–shell structure found in nature has been widely imitated to produce lightweight, stiff structures, such as surfboards and rotor blades. Before the widespread adoption of additive manufacturing, wire-woven, three-dimensional structures were developed for the cores of ultralight sandwich panels [28]. A historically and technologically important class of such materials is the kagome lattice [29]. Advances in fabrication techniques have enabled the creation of materials with complex topologies optimised for specific needs, such as thermal expansion [19], vibration control [30], and acoustic attenuation [31].

A first general review of cellular materials, including quantitative models for the properties of cellular solids, was provided by Gibson & Ashby (GA) in 1987 (with a second edition in 1997 [4]). Based on a few earlier publications on the mechanical properties of honeycombs [32,33,34,35], they developed a series of precise approximations for the elastic constants of hexagonal honeycombs constructed from linear elastic materials, assuming that the walls are subject to bending loads.

Likewise, under the same assumptions, they provided an expression for the elastic buckling of the walls and their plastic collapse. The assumptions used for the collapse of brittle honeycombs under compression and for fracture under traction are open to discussion; nonetheless, they yield closed-form equations that provide reasonable approximations. Equations for viscoelastic deformation were also derived. The authors considered nonlinear and rubber-like materials, but these are not amenable to closed-form solutions.

Contrary to 2D structures, their chapter on 3D cellular solids (3D-CS or foams), GA starts by pointing out errors in the literature and the limitations of full 3D modelling of cellular structures under any given stress state. With commercial finite element software hardly emerging at the time of their publication, they rely on a rather arbitrary generic unit cell to try to capture the general laws ruling the relationship between the properties of the material used to construct the foam, the relative density of the foam, and the mechanical properties of the foam, considered as a homogeneous medium.

Using dimensional analysis and the basic equations for the bending of Euler–Bernoulli beams [36], they expressed the relationships between these quantities in terms of power laws [37] in the style pioneered by Ashby [38] (Section 2.3). For example, one can consider, in a generic sense, a property $P_{C}$ of the cellular solid, considered as a homogeneous material. $P_{C}$ can be any of the elastic constants involved, the yield strength, a buckling limit, or brittle collapse strength. For viscoelastic phenomena and creep, $P_{C}$ may depend on time. The corresponding property of the constitutive material forming the walls or trusses of the material is indicated as $P_{S}$. In cases where such a property does not exist, e.g., for the buckling limit, an equivalent property is used, such as to render the left-hand side of Equation (1) dimensionless:

(1) $$\frac{P_{C}}{P_{S}}=C{\left(\frac{\rho_{C}}{\rho_{S}}\right)}^{n}f\left(\Gamma \right)$$

Here, $\rho_{C}$ and $\rho_{S}$ are the apparent density of the cellular material and the density of its solid constituent material, respectively. The relative density $\rho_{R}=\frac{\rho_{C}}{\rho_{S}}<1$. C is a constant that depends on the geometry of the beams or walls (thickness, radius, length, and spatial arrangement) and their predominant deformation mode (bending or stretching). The exponent n depends on the deformation mode and the character of $P_{C}$. In the Ashby approach [38], its value is an integer or a simple rational number. $f\left(\Gamma \right)$ refers to geometric effects that cannot be fully accounted for in a purely dimensional analysis.

While alternative theories for cellular materials were developed contemporaneously with GA, such as the minimum solid area [39] and the Zhu–Knott–Mills model [40], GA has been used most extensively to describe 3D cellular solids (3D-CS), as reviewed by Zhong et al. [41]. The model requires slender trusses, defined by a diameter-to-length ratio of l/d ≥ 5. Contrary to some results on honeycombs, the GA model for 3D-CS does not require the full solution of the hyperstatic mechanical equilibrium problem in the unit cell. This means it is not predictive since C must be calibrated experimentally. Due to the complex geometry and mechanical behaviour of the nodes between the trusses, n becomes a fitting parameter.

This approach has been illustrated by Al Ketan et al. [42], who produced an extensive series of unit-cell-based materials via laser powder bed fusion (LPBF) of maraging steel. Kladovasilakis et al. [43] applied the GA model to a broad set of literature data for Young’s modulus, confirming the power-law trend. Their results also show a wide data cloud around the general trend, indicating that differences in geometric details and manufacturing procedures affect the values of C and n in a way that the GA cannot capture. Extensions to the GA approximation were reviewed by Iqbal and Kamiński [44].

Some articles report substantial deviations between the Gibson–Ashby model and experimental data [45,46]. Zhong et al. [41] noted that GA exhibits deviations of up to 300% for additively manufactured metal lattices, as most of them comprise trusses  l/d < 5, which contradicts the model basis. These authors extend the GA approach by employing Timoshenko beams [47] within a finite element framework using 3D beam elements. Hence, they can account for shear and torsion in addition to the more elementary bending or stretching-dominated configurations used earlier.

Based on the papers cited in this introduction, there is little doubt that the work by Gibson and Ashby is the single most important leap in the design of CS. However, more precise analytical and numerical models are now available due to advances in computational power and calculation methods over the past 40 years, together with the capability to produce almost any geometry through additive manufacturing.

In the following, the authors analyse a broad selection of papers that illustrate the general trends in modelling the mechanical properties of CS for use as a structural material in lightweight components. The important topic of postyielding behaviour, which determines energy absorption, is not covered to keep the review within reasonable limits. Analysis of mechanisms, as opposed to structures (Section 2.4), is kept to a minimum. The review focuses on stiffness and strength.

The review was performed to answer a very specific question confronted by the authors: if an engineering team decides to use lightweight high-strength cellular solids within a proposed mechanical design, what are the tools to select and optimise such structures without recurring to extensive experimental optimisation or lengthy schemes of numerical simulation?

As such, a systematic meta-analysis to quantify the leading trends or the number of papers in each of the sub-fields is not the goal of this work. Instead, the review is based on an iterative sieving and refinement of the information until the above question can be answered for a broad range of materials and geometries.

A first selection consisted of broadly cited review papers and foundational works since GA. This forms the basis for the introduction and Section 2. A second iteration was made to find more specific examples of the materials covered in the review papers, focussing on material classes and geometries which are either overlooked in the general reviews or are exceptionally promising in terms of lightness, stiffness or strength.

For the subsequent iterations, covering Section 3, Section 4, Section 5 and Section 6, additional papers were selected that refine the information about specific modelling strategies. The study was purposedly biased against purely experimental works, analyses of a single structure under a single loading mode, and semi-empirical models, i.e., models that require extensive experimentation for their calibration. Exceptions were made for works that fill the gaps in terms of materials selection and geometry or high-quality experimental papers which highlight the need for more advanced and generally applicable modelling strategies.

Section 2 presents an overview of classification schemes for CS. Section 3 reviews 2D models as approximations to honeycombs and 3D materials. Section 4 analyses the literature on 2.5D materials, i.e., honeycombs and sandwich panels. Section 5 addresses the broad field of 3D structures. A brief overview of the use of artificial intelligence (AI) for predicting mechanical properties is given in Section 6. The paper concludes with a critical analysis of the current state of the art and future directions in the field.

## 2. Classification of Cellular Materials

### 2.1. Criteria

The different criteria and methods for classifying CS present a surprisingly broad spectrum, strongly influenced by research focus across fields. Mechanical [3,4,48], thermal [49,50], fluid [1,51], chemical [52,53,54], biological [55,56,57], and biomedical [2,58,59] materials will partially determine how researchers classify their subject. Zok et al. [60] even proposed a lexicon, grammar, and syntax to describe 3D truss structures, which represent only a fraction of all cellular materials described in the literature.

**Figure 1:**
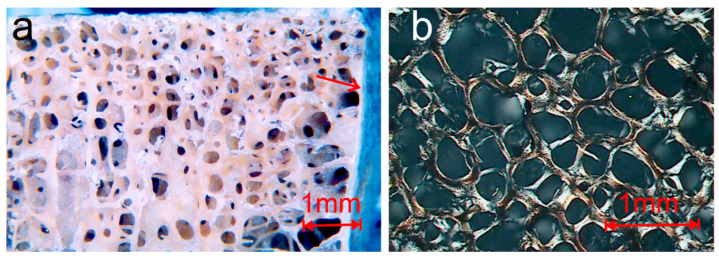
(**a**) Vertical cut through a porcine vertebra. The arrow indicates a thin layer of cortical bone surrounding the cancellous bone, which exhibits a poorly defined columnar arrangement, resulting in anisotropy. (**b**) Thin horizontal section showing more detail of the stochastic, transversely isotropic structure.

The materials that constitute the CS can be divided into metals, polymers, and ceramics. Composite materials are found in the cell walls in wood and the trusses of cancellous bone, with the former consisting of a matrix of amorphous cellulose reinforced by hemicellulose and lignin fibres (all polymers) [56] and the latter built from hydroxyapatite (a ceramic) toughened by collagen fibres (a polymer) [59]. Wood and bone grow naturally, and the latter can remodel its structure as a function of loading conditions during life [61,62] (Figure 1).

**Figure 2:**
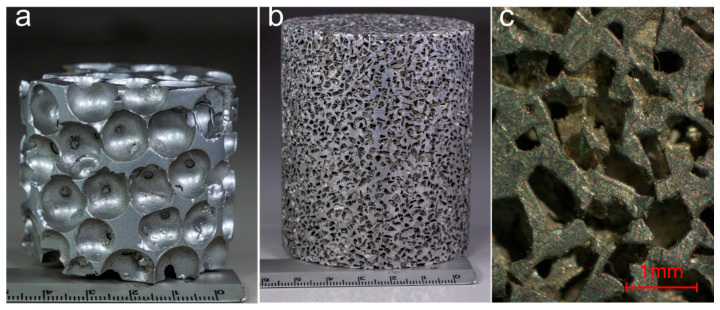
(**a**) Aluminium foam produced by the infiltration of a preform of hollow spheres. (**b**) Aluminium foam produced by the infiltration of coarse salt grains. (**c**) Detail of (**b**).

On the other hand, engineering materials can be classified by their production techniques. Investment casting of low-melting alloys was used in early experiments [63,64]. Additive manufacturing provides a versatile method to produce 3D-CS [65,66]. Replication casting [67,68,69,70,71], infiltration of preforms made from hollow spheres [72,73] (Figure 2) and the use of foaming agents inducing gas bubbles during solidification will produce random or pseudorandom CS [74,75]. Self-foaming can also be achieved through controlled volume contraction resulting from phase transformation during heat treatment [76,77]. Foaming is also widely used for polymers, either by H_2_O or CO_2_ formed during the polymerisation reaction or by injecting gas during the process [13,14,78]; ceramic foams can likewise be formed by gas evolution in the semisolid state [79,80,81,82].

Additive manufacturing provides a versatile and cost-effective method for producing 3D components [65,66]. Partial sintering of low-density powders results in random structures with open cells [83,84,85], but sintering can also be applied to dried stabilised foams [86] or slurries deposited on polymer templates, which are calcined during the process [87]. The use of spacer materials enables the production of sintered materials, such as those used in replication casting [88]. The sintering of hollow spheres has been used to design advanced ceramic foams [89]. Filament fusion can also be employed to produce green forms for ceramic and metallic structures with a finely controlled geometry [90]. Infiltration casting of templates produced from biomaterials permits an exact replication of their structure [91].

Corrugation and punching of metal sheets have been widely used to produce commercial regular cellular materials and honeycombs [92]. An extension of these relatively simple techniques is the development of origami-inspired metamaterials [48,93]. Stacking of the corresponding structures allows their use to be extended from classical sandwich panels to fully 3D-CS. Truss-based materials and metamaterials can be produced through wire weaving [28], enabling the manufacture of both 2.5D and 3D structures. These techniques are characterised by their reliance on centuries-old metal-forming and joining methods.

This contrasts with AM techniques, which have a history spanning a few decades and are still in their early stages of development. Filament extrusion [94,95,96], laser powder bed fusion [22,42,43,97], and digital light processing [98,99,100] are examples of some important techniques in this field and allow the production of periodic cellular materials with a broad range of unit cells, which will be discussed in Section 2.3.

In the following, CS for mechanical applications will be covered, classified first by the dimensionality of the structure (1.5D, 2D, 2.5D, 3D, 4D), followed by its degree of regularity (random, periodic, and gradient). Most of these geometries can be obtained by a broad range of production processes and using different material classes [101,102]. A summary of the possible combinations of geometry, materials and manufacturing processes will be provided in Section 6.

### 2.2. Basic Geometry

The study of classical mechanics is limited to three dimensions. Engineering materials always occupy a volume; hence, they only exist in 3D. Nonetheless, simplified models of some CS can be expressed in one dimension (1D) [103] and two dimensions (2D) (Section 3). A material where the radius is much smaller than the length, such as chains and fibres, will be classified as 1.5D. These are not further considered in this review. Platelike structures, particularly sandwich panels, are commonly classified as 2.5D (Section 4). For many applications, their out-of-plane properties are much more important than the in-plane behaviour of the pure sandwich core. The latter can often be modelled conveniently in 2D.

The meaning of 3D (Section 5) is self-evident but generally requires that a reasonable number of basic structural units (random or periodic) are contained in each dimension of the structure. For example, a panel with two structural elements in the thickness dimension is still often considered to be 2.5D [28]. An early example was analysed by Hutchinson et al. [29]. Complex nonlinear effects, such as rubber-like behaviour and shape memory [104,105], used in 4D materials require advanced material models, which will not be reviewed here.

At the mesoscale, a distinction is made between periodic, random, pseudorandom, and gradient structures. Periodic structures are formed by the repetition of a unit cell by translation over an entire multiple of the lattice parameters. They will be discussed in Section 2.3. Randomness can be defined as a set of probability density functions that describe the size and shape of cells, walls, or trusses. The corresponding plane or space-filling structure is then the result of a random process.

One of the best-known random processes is the Poisson point process, where the coordinates of the points are obtained by uniform and independent sampling over the volume of the material. Cellular structures can be defined by using the Voronoi [106] or Delaunay tessellations [107] over this point process. The resulting Poisson Voronoi tessellation produces microstructures that may deviate significantly from microscopic observations [108]. The Centroidal Voronoi Tessellation [103,109,110] and Laguerre–Voronoi [111,112,113] can be used to produce microstructures that are either more homogeneous than the Poisson Voronoi tessellation or that contain a controlled degree of heterogeneity.

Pseudorandom structures are based on the repetition of a unit cell with superimposed random variations [114] or by the regularisation of a Poisson Voronoi tessellation [115,116]. Variations may also refer to the thickness of trusses and walls. The presence of defects, either by the production techniques in experimental settings [21,117] or numerically in modelling [118,119,120], can introduce randomness in an otherwise periodic structure. Gradient structures can be developed by varying the material density through modifications to the wall thickness or cell size [121,122] or by altering the unit cell across different layers of the material [123].

At the microstructural level, a distinction can be made between open and closed cells. This concept is closely related to the distinction between accessible and non-accessible porosity, as defined in chemistry and geosciences, which indicates whether a fluid can percolate through pores. Both types of porosity (open and closed cells) can be present in the same material. The cell walls of closed cells can contribute significantly to the strength and stiffness of a material compared to its open-cell equivalent [4,124].

The interaction between the load-bearing capacity of the solid and the behaviour of the fluid contained within the cells is studied in the field of poroelasticity [125,126]. Liquid-filled closed-cell media will inherit the incompressibility of the liquid phase, and the fluid pressure may modify the strain mode in the wall [127]. Gas-filled pores can be modelled using the ideal gas law [128]. The contribution of the gaseous phase is often neglected in modelling studies [129,130,131]; however, it has been noted that the analysis of a fluid-filled cavity is included in some commercial FEM packages [132,133]. The reported modelling results differ significantly from those obtained without considering the fluid.

Fluid behaviour in open-cell structures at low Reynolds numbers is described by Darcy’s law [134], which states that the flow velocity is proportional to the pressure gradient in the medium. The proportionality constant is called the hydraulic conductivity. It is inversely proportional to the dynamic viscosity of the fluid and strongly affected by the cell size and tortuosity of the fluid path in the structure. Poroelastic behaviour is fundamental to understanding fluid-filled biological cellular materials [135,136,137].

### 2.3. Unit Cells

**Figure 3:**
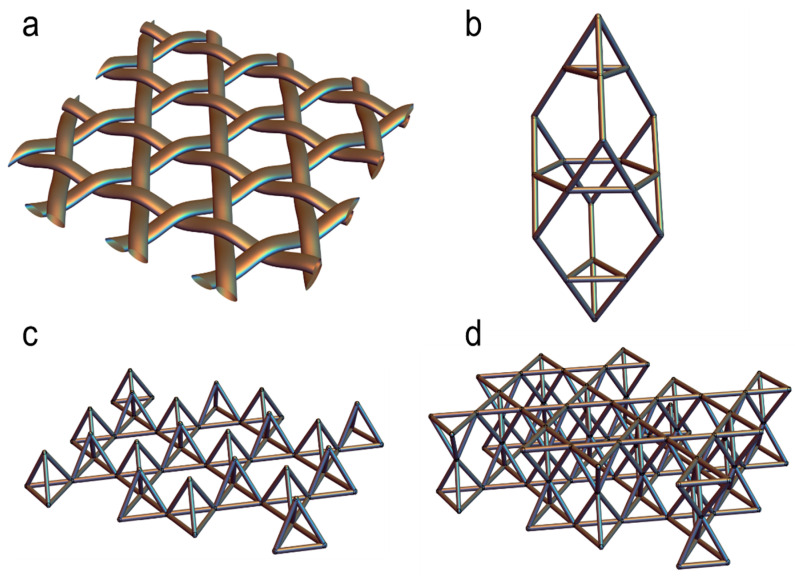
(**a**) Traditional kagome weaving pattern, (**b**) rhombohedral unit cell of a 3D kagome lattice with the vertical axis perpendicular to the plane of the pattern, (**c**) single-layer 2.5D kagome honeycomb, and (**d**) bilayer kagome honeycomb.

Regular lattice structures imply the concept of a unit cell. Although this concept is rigorously defined by crystallography, with 7 crystal systems, 14 Bravais lattices, 32 point groups and 230 space groups, this classification is not generally used in the study of cellular solids. Most studies focus on the many variants of the cubic crystal system and are limited to adding beams and walls between the lattice points in the several Bravais lattices. Many of these cubic lattices can be transformed into other symmetry classes by applying an affine transformation [60,114,127]. Zok et al. [60] present a 3D kagome structure with rhombohedral symmetry (Figure 3).

It is not within the scope of this review to provide a list of all cubic unit cells that have been modelled in the literature [21,22,41,48,138]. Several studies have searched for structures that exhibit the highest possible isotropy [139]. An important aspect of composite design is the use of anisotropy to create materials that are optimally adapted to specific loading conditions. Nevertheless, there are clear examples [140] where the anisotropy of the lattice is so strong that only a slight deviation from the ideal loading conditions would cause a drastic reduction in strength and stiffness.

The benefit of anisotropy is illustrated by biomaterials, which can adapt their structures as a function of loading. Because a stress tensor has three perpendicular principal stresses, biomaterials tend to be orthotropic or transversely isotropic in nature. Transversely isotropic materials can be approximated by using hexagonal unit cells in additive manufacturing, and the hexagonal honeycomb is widely used in sandwich panels. Triply periodic minimal surfaces (TPMS) with tuneable anisotropy were recently proposed by Daynes [141].

Historical preferences also influence the choice of unit cells for modelling in the various sub-fields of study. The so-called Gibson–Ashby cell, initially used to explain the basic concepts of dimensional analysis for cellular solids, has begun to take on a life of its own [42]. However, it was originally intended solely as a generic calculation example with no practical applications. Kelvin cell foams [40] are widely used in the study of polymeric [114,127,130,142] and metallic foams [129]. At the same time, the octet truss lattice is prominent in the field of additively manufactured cellular solids due to its low anisotropy and high stiffness-to-weight ratio [122,140,143].

A final distinction to be made is between truss-based and shell-based structures. Closed-cell materials can be developed by adding walls to the cubic cells mentioned above. However, there are additional examples, such as hollow-sphere packing [143], TPMS, and origami materials [48,105,144], where the latter two can sometimes be loosely connected in terms of the geometry of their unit cells [145]. An overview of the historical development of TPMS, from a geometric viewpoint, was provided by Schoen [146], and their symmetry classes have been studied in detail [147].

**Figure 4:**
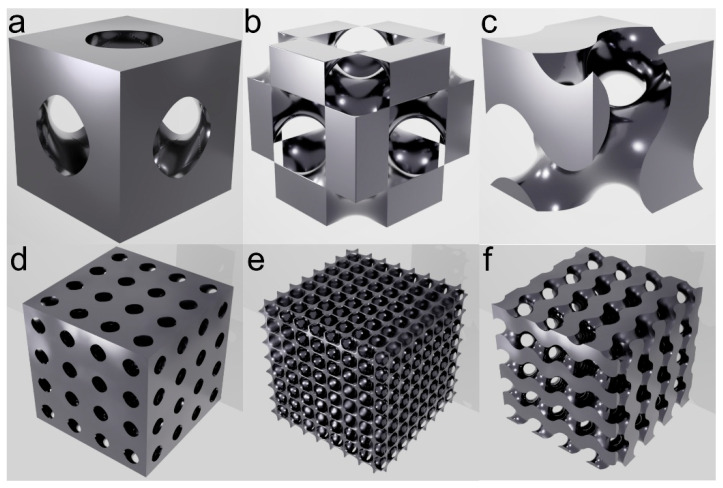
Skeletal TPMS. (**a**) Schwarz primitive unit cell; (**b**) Schwarz diamond unit cell; (**c**) Gyroid unit cell. (**d**–**f**) 64-cell structures based on (**a**–**c**).

**Figure 5:**
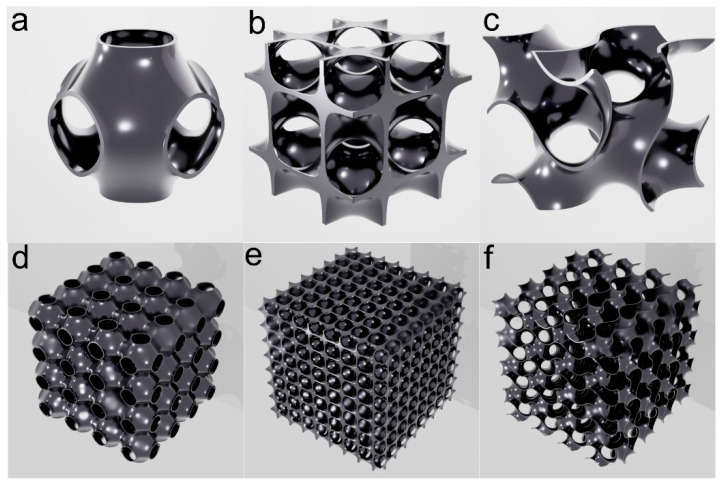
Sheet TPMS. (**a**) Schwarz primitive unit cell; (**b**) Schwarz diamond unit cell; (**c**) Gyroid unit cell. (**d**–**f**) 64-cell structures based on (**a**–**c**).

A TPMS, in the geometric sense, is a continuous 2-manifold with mean curvature equal to 0 everywhere, embedded in 3D space. It divides the space into two disjoint, contiguous volumes. The content of the unit cell can be defined either by one of these volumes [148] or by assigning a thickness to the surface itself [149], sometimes indicated as skeletal and sheet TPMS. TPMS locally has the least area of all surfaces in a neighbourhood bounded by the same curve. Therefore, they are of interest for applications that require a high specific surface area, such as resorbable scaffolds for biomedical implants [150,151], heat exchange [149,152], and catalysts [153]. Some examples are shown in Figure 4 and Figure 5.

### 2.4. The Maxwell Stability Criterion/Bending vs. Stretching

For pin-jointed trusses or structures where joint stiffness can be reasonably neglected, the only mechanism for load transfer within the truss is tension–compression. Under such conditions, buckling appears as a possible failure mechanism, in addition to brittle fracture or plastic yielding. If, on the other hand, the joints are stiff, bending will predominate the deformation behaviour, as was the case for the Gibson–Ashby cell. The differences between bending-dominated and stretching-dominated unit cells were clearly outlined by Deshpande et al. [154]; a recent review was provided by Zhong et al. [41].

**Figure 6:**
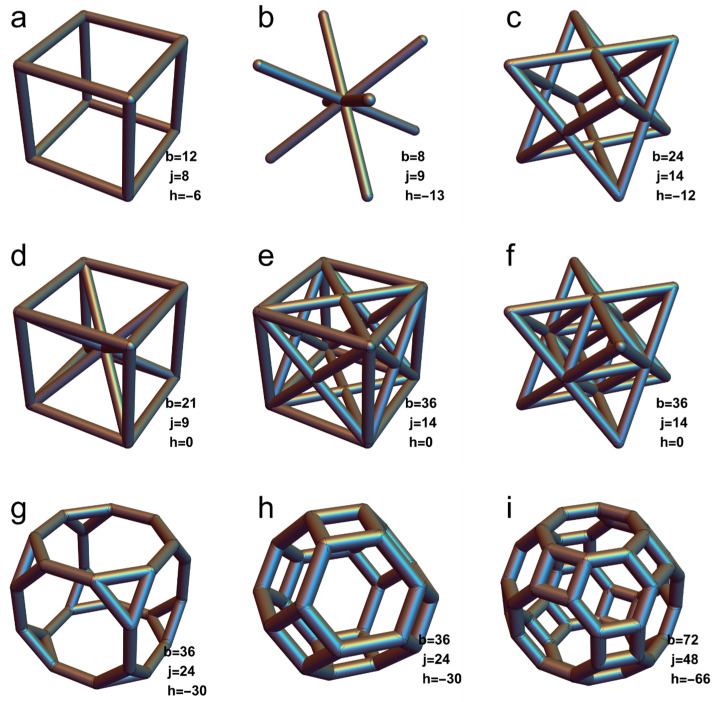
Truss-based unit cells. (**a**) Simple cube. (**b**) Body-centred (BCC) diagonals. (**c**) Face-centred cube (FCC) diagonals. (**d**) Complete BCC, a combination of (**a**,**b**). (**e**) Complete FCC, combination of (**a**,**c**). (**f**) Octet truss obtained from (**b**) by adding the central octahedron. (**g**) Truncated cube. (**h**) Truncated octahedron. (**i**) Truncated cuboctahedron.

In a slightly simplified form, the distinction is given by the Maxwell criterion (see ref. [155] for the general form) by defining a parameter *h*:

(2) h=b−2j+3

(3) h=b−3j+6

where *b* is the number of trusses (beams) and *j* is the number of junctions. Equation (2) is valid for 2D, and Equation (3) is valid for 3D. The value of h can be interpreted as the degree of hyperstaticity, with h=0 corresponding to an isostatic structure (i.e., the number of equations equals the number of variables), and h<0 indicating a mechanism. Some truss-based cubic unit cells are presented in Figure 6. Increasing the value of h ≥ 0 increases the stiffness of the structure. For h>0, the displacements and forces in the structure can only be solved by introducing additional equations accounting for the deformation of the trusses.

The first row corresponds to highly anisotropic structures [139,140], which can derive stiffness only from their junctions as h<0. Combinations of these produce the FCC and BCC models h = 0, which also hold for the octet-truss structure. More advanced unit cells, corresponding to some of the Archimedean solids, are the truncated cube, truncated octahedron, the truncated cuboctahedron, and the tetrakaidecahedron or Kelvin cell, which have h≪0. Adding all the longest diagonals to all faces converts these faces into rigid plane structures. It can be verified that h=0 in this case.

If a pin joint between $n_{j}$ beams is substituted by a stiff joint, $3\left(n_{j}-1\right)$ equalities are added due to the imposed equality of the rotational degrees of freedom for each beam. If all joints are stiff, the structure is always hyperstatic. Then, h<0 defines bending-dominated materials and h≥0 corresponds to stretching-dominated materials. Stretching-dominated materials are much stronger and stiffer than bending-dominated materials for the same relative density [154]. Structures with excessive anisotropy [140] are generally bending-dominated. General parametric formulations for the stiffness and strength of both types of materials are given in Table 1.

Table 1 must be interpreted with caution for practical CS. Pin joints are rare, although they may be present in certain honeycombs produced by the slotting method. Honeycombs are tempting subjects of study, as they are accessible to relatively simple 2D solid mechanics calculations [156,157,158] and are predominantly bending-dominated. However, they are generally not meant to be used free-standing. The addition of a face sheet removes the displacement degrees of freedom at the edges of the structure, inducing a stiffness that is much higher than that predicted by simple 2D calculations [30,159,160,161,162], as is the case for sandwich panels with a bending-dominated wire-woven core [163]. Finally, the concept of bending-dominated closed-cell structures must be taken cautiously, as Cauchy’s theorem states that a polyhedron with rigid faces is rigid; hence, as the fraction 1−ϕ of material in the walls increases, the structure will become hyperstatic.

**Table 1:** Approximate estimations for the strength and stiffness of different cell architectures [164,165]. ϕ is the fraction of material contained in the edges compared to the total solid fraction.

Stiffness	Bending-dominated		Stretch-dominated	
Open	$\frac{E_{C}}{E_{S}}=C_{1}{\left(\frac{\rho_{C}}{\rho_{S}}\right)}^{2}$	(4)	$\frac{E_{C}}{E_{S}}=C_{3}\left(\frac{\rho_{C}}{\rho_{S}}\right)$	(5)
Closed	$\frac{E_{C}}{E_{S}}=C_{1}{\left(\phi \frac{\rho_{C}}{\rho_{S}}\right)}^{2}+D_{1}\left(1-\phi \right)\frac{\rho_{C}}{\rho_{S}}$	(6)	$\frac{E_{C}}{E_{S}}=C_{3}\left(\frac{\rho_{C}}{\rho_{S}}\right)+D_{1}\left(1-\phi \right)\frac{\rho_{C}}{\rho_{S}}$	(7)
Strength	Bending-dominated		Stretch-dominated	
Open	$\frac{\sigma_{yC}}{\sigma_{yS}}=C_{2}{\left(\frac{\rho_{C}}{\rho_{S}}\right)}^{3/2}$	(8)	$\frac{\sigma_{yC}}{\sigma_{yS}}=C_{4}\left(\frac{\rho_{C}}{\rho_{S}}\right)$	(9)
Closed	$\frac{\sigma_{yC}}{\sigma_{yS}}=C_{2}{\left(\phi \frac{\rho_{C}}{\rho_{S}}\right)}^{\frac{3}{2}}+D_{2}\left(1-\phi \right)\frac{\rho_{C}}{\rho_{S}}$	(10)	$\frac{\sigma_{yC}}{\sigma_{yS}}=C_{4}\left(\frac{\rho_{C}}{\rho_{S}}\right)+D_{2}\left(1-\phi \right)\frac{\rho_{C}}{\rho_{S}}$	(11)

Likewise, the stiffening of junctions can increase stiffness and strength far beyond what is predicted for bending-dominated structures [28,124,166,167]. For stretching-dominated structures, the role of filleted joints is less clear. Depending on the material and manufacturing method, an increase in strength has been reported for stiffened joints, resulting from a reduction in local stress concentration at the joints [168]. In contrast, an increase in buckling risk has the opposite effect [169,170]. At a given apparent density, the transfer of material from the trusses to the joints will weaken the former and strengthen the latter. Except for the lowest-density structures, stiffening of the joints generally produces favourable effects in acrylic-based honeycombs [171]. Within the vast body of literature on truss-based CS, this topic is clearly underrepresented.

## 3. 2D Structures

### 3.1. 2D Models for 2.5 and 3D Structures

Honeycombs have been widely studied for use in lightweight structures. Fan et al. conducted an analytical study of 2D honeycombs [157] considering the five types of mechanical behaviour proposed by GA [4]: linear elastic deformation, elastic buckling, plastic collapse, brittle failure and fracture. Tankasala et al. [158] obtained elasto-plastic yield surfaces of an incompressible, filled hexagonal honeycomb using slender beam theory. Schiantella et al. [172] investigated the onset of yield using upper-bound analysis for single and multiple cells in 2D honeycombs, with a particular focus on the effects of imperfections and on different geometries. An extensive list of approximate and analytical results for the in-plane properties of advanced honeycomb geometries was provided in the review by Qi et al. [156], who studied advanced designs to improve the mechanical properties of honeycombs. Hierarchical plane truss structures with rigid joints can be analysed systematically using a matrix-based approach inherited from structural mechanics [173].

**Figure 7:**
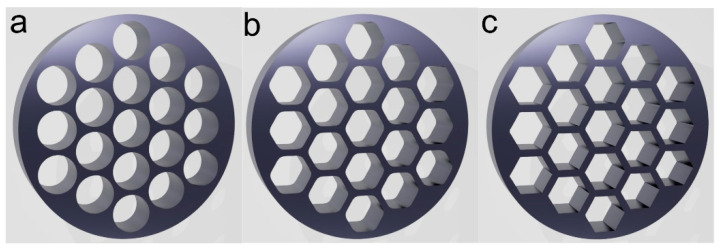
(**a**) Circular honeycomb. (**b**) Rounded hexagons. (**c**) Regular hexagons. All geometries are produced as a tiling based on Gielis’ superformula [177,178] with hexagonal symmetry and different shape parameters.

The most common technique to predict mechanical properties is the finite element method (FEM). Ronan et al. [174] tested 2D honeycombs and lotus root materials (Figure 7) under tensile strain using FEM to model individual cells, comparing the results with those from a mesh of cells with missing cell walls. Restrepo et al. [175] simulated compression tests for 2D honeycombs and kagome cells with regular deformed shapes. Iltchev et al. [176] studied circular honeycombs as a homogeneous equivalent medium (HEM) to reduce computational costs. This technique requires the accurate characterisation of the relationship between microscopic and macroscopic stresses and strains. Uniaxial, simple shear and biaxial tests were simulated, and yield surfaces were presented.

Liu et al. [179] conducted FEM simulations of the yield point for a standard specimen in 2D stress space to obtain yield surfaces of honeycomb meshes of different sizes by the equivalent plastic strain increment approach. Yielding was defined as the increment at which the equivalent plastic stress equals the yield stress at any point in the specimen. Karri and Kambagowni [180] recently presented an extensive finite element study based on cubic unit cells with face sheets. This allowed them to calculate the elastic anisotropy of an extensive set of candidate materials. In addition to the significant variation in stiffness, strength, and energy absorption across distinct unit cells, they highlight the importance of the face plates and stress concentrations at the truss joints.

Su et al. [181] tested auxetic re-entrant honeycombs with different off-axis angles. The off-axis angle affected the anisotropic properties and Poisson’s ratio. A pressure-dependent, anisotropic yield criterion was proposed to capture the multiaxial in-plane yield behaviour. Lu et al. [182] studied advanced tetrachiral honeycombs using FEM and experimental methods, focusing on optimising the structure by varying its geometric design parameters. A computationally efficient approach was shown by Ni et al. [183,184], who modelled the spring-like cantilevers in their auxetic structures with shell elements.

### 3.2. Random Honeycombs

Most studies of random honeycombs use FEM. Alkhader et al. [185] obtained stress–strain curves for compression and tensile tests at different levels of irregularity in honeycomb, tetragonal, and triangular structures. Sotomayor et al. [116] obtained stress–strain curves for compression tests with different irregularities. Mukhopadhyay et al. [186] obtained elastic moduli by closed-form solid mechanics calculations for regular auxetic honeycombs and by FEM for lattices with spatial irregularity.

Multiple studies of random 2D honeycomb structures were proposed using Voronoi cells [116,185,187]. It was found that the loss of periodicity may increase bending moments at joints, shifting the structural response toward a bending-dominated response. This decrease in the fraction of axially load-bearing trusses leads to a moderate reduction in both macroscopic stiffness and strength. Sotomayor and Tippur [116] used a regularised Poisson process [188] for the nuclei of Voronoi cells [186] and concluded that a fully irregular Voronoi honeycomb was approximately 66% stiffer than its regular counterpart.

Auxetic honeycomb structures with different perturbation parameters were tested by analytical and FEM methods [181,186,189]. The irregularity changed its relative density, and Poison’s ratio resulted in a less negative value, impairing the specific properties for which this structure is designed. Mizzi et al. [190] studied stochastic hexachiral honeycombs in the presence of translational disorder. They found that this geometry is tolerant of retaining its original Poisson’s ratio despite 90% disorder, unlike auxetic honeycombs.

Liu et al. [191] generated a bioinspired stochastic honeycomb structure with two levels of randomness, in which the first-level structures show random wall thickness, and the second-level structures have circular pores of different sizes. Through case studies, they demonstrated that the strength-to-weight ratio of the honeycomb can be enhanced by modifying the pore distribution within the walls. Random honeycombs based on cancellous bone geometry, with random removal or thinning of trusses, were studied by FEM to simulate the effect of bone disease [120], showing that truss removal was more deleterious than truss thinning for the same amount of material removed.

Defects in honeycombs significantly affect their mechanical performance. Ajdari et al. [192] studied regular and random honeycombs with missing walls using FEM. The yield strength of the cellular structure decreases by more than 60% when 10% of the walls are missing in both random and regular honeycombs. Ronan et al. [174] tested 2D honeycombs and lotus materials with missing cell walls. An array of missing cell walls generates a crack-like irregular cell. Large inclusions within the honeycomb induce a local strain concentration, thereby further degrading the ductility of the material.

Restrepo et al. [175] simulated compression tests for 2D honeycombs and kagome cells with broken and missing walls. Schiantella et al. [169] studied the effect of imperfections for different geometries: hexagonal, rhomboidal, triangular and kagome. Distributed defects weaken the lattice, particularly in stretch-dominated structures such as triangular and kagome configurations. Conversely, localised variations indicate that concentrating defects in limited domains has a more pronounced effect on the lattice strength. The kagome structures are most strongly affected by defects.

## 4. 2.5D Models and Sandwich Panels

### 4.1. Honeycomb-Based Sandwich Panels

Honeycomb sandwich structures combine high flexural rigidity and bending strength with low weight. Fan et al. [157] reported an analytical study on the in-plane behaviour of sandwich trusses (isogrid and honeycombs) and the out-of-plane buckling of the structure. They used the result to analyse the behaviour of a honeycomb produced with sandwich trusses. They found that the hierarchical honeycomb is much stiffer and more damage-tolerant than a simple honeycomb at the same apparent density.

Iltchev et al. [176] modelled sandwich structures with a tube-stacked core as a homogeneous equivalent material (HEM). The properties of the HEM under triaxial loading were determined using FEM and utilised for a full-scale simulation of the sandwich structure. The results of the HEM-based simulations are comparable to those of full-scale modelling, with lower computational costs. A remarkable aspect of this study is that the sandwich plates are parallel to the tube walls, rather than the more common perpendicular configuration.

Hussain et al. [193] modelled a honeycomb sandwich structure for fatigue simulations, evaluating specimen life based on the load–displacement response. Xiao et al. [194] developed an FEM model to investigate the dynamic deformation evolution of two face sheets with an auxetic re-entrant honeycomb core. Ha et al. [195] proposed a bioinspired honeycomb sandwich panel based on the microstructure of a woodpecker’s beak. The panel showed superior energy absorption (125% more) compared with a conventional honeycomb sandwich panel.

Thomas and Tiwari [196] presented a review of the crushing behaviour of honeycombs. They focus on the out-of-plane response of sandwich panels but largely neglect the strengthening effect of the face sheets. A systematic study of this effect was presented by Wei et al. [197] for the in-plane crushing of sandwich columns, but they provide limited information about modelling such effects. Their failure mechanism map is entirely based on experiments [198]. A review on the optimised design of sandwich structures, with a focus on core-sheet synergy, was provided by Sahu et al. [199]. This review pays no attention to modelling. Corrugated cellular solids were modelled in 3D by Choi et al. [200], showing the importance of wall buckling and collapse but again neglecting the effect of the face sheets.

### 4.2. Kagome Structures

The kagome lattice consists of the vertices and edges of the trihexagonal tiling. Hyun et al. [201] analysed a kagome core unit cell under compressive and shear loading. The kagome cell is more resistant to plastic buckling and has a greater load capacity than the tetragonal core in both cases. Their study did not account for the face plates. Park et al. [200] extended this analysis to dynamic external loads, with the same limitations. As imperfections in the trusses were added, the impact energy transfer decreased, and the vibration magnitude increased. Optimally designed kagome structures with strategically implemented defects can be used to develop efficient energy-absorption materials.

The wire-woven bulk kagome (WBK) is a multilayered truss-type cellular metal introduced by Lee et al. [202] based on systematic assembly of helical wires in six directions. Lee and Kang [203] tested the geometry in compression using two different wire materials. Later, Kang [124] obtained analytic solutions based on detailed structural mechanics calculations for compression and compared them with FEM and experimental results. The WBK fabricated using spring steel wire showed excellent strength and low anisotropy [204]. Feng et al. [205] combined an experimental and theoretical study of the thermomechanical properties of WBK by compressive loading. Brazing the joints increases the core strength by more than ten times. Kim and Kang [206] composed an actuator of a WBK truss using a shape memory alloy. The actuator was physically tested, and an analytical solution was derived based on buckling mechanics.

### 4.3. Origami Structures

Origami is a Japanese technique of folding and assembling planar material to create three-dimensional shapes whose variety and complexity depend on the number, order and orientation of the folds [48]. Origami is used to design ultralight, customizable mechanical materials [93,207,208,209,210]. Origami models can be classified further into a relatively small number of interconnected spherical mechanism combinations [210]. Origami networks include single loops (traditional square twist [211]), 1D periodic structures (Schafer’s blinking eyes [212]), 2D periodic structures (Miura-ori pattern [213]), and nonperiodic structures (Shafer’s monster mouth [210]). Origami design is based on a plate and hinge mechanism. They offer enhanced flexibility, deformability and compactness due to their properties coupled with a dynamically alterable folding pattern [214].

Most origami-inspired mechanical material designs are based on the Miura-ori folding pattern [213,215], which has a single degree of freedom. Their mechanical properties as a metamaterial were analysed by Schenk and Guest [208], showing that the folded shell structure has a negative Poisson’s ratio for in-plane deformations and a positive Poisson’s ratio for out-of-plane bending. Other origami structures with multiple degrees of freedom can be used to design highly flexible and deformable 3D structures [216].

Silverberg et al. [93] discovered that a Miura-based mechanical material with tuneable stiffness is technically feasible by introducing a reversible pop-through defect. Harris and Mc Shane [217] tested origami specimens made from Miura-folded sheets using tensile, compression, and hardness tests, as well as FE analysis. The nonperiodic Ron Resch pattern [218,219] is a series of origami tessellations formed by the insertion of a star-like folded tuck. This pattern demonstrates a good load-bearing capability under an axial compressive force [219]. Materials with higher-order symmetry have an even greater load-bearing capacity. Their calculation of the mechanical properties is mainly based on a geometric analysis of the degrees of freedom of the structure and does not include solid mechanics calculations.

The rare square twist pattern requires two different modes of deformation, creasing and facet bending. Creasing represents a plastic mode of deformation, whereas bending is reversible. The resulting mechanical properties have been studied from geometrical considerations and mechanical tests [211] without modelling the structural mechanics of this class of materials. An indication of the limited extent of modelling studies in this field is that several important reviews on the mechanical properties of origami materials hardly cite any theoretical efforts and focus on limited sets of mechanical tests [105,220].

## 5. 3D Structures

### 5.1. Periodic Truss-Based Cellular Solids

Finite element models for truss-based cellular solids are abundant, with emphasis on defects and residual stresses. Moussa et al. [221] studied tetrahedron structures with induced defects and their impact on mechanical properties. The octet truss lattice is one of the most studied geometries. Generally, the stress state is limited to uniaxial compression [42,222,223], and the reported properties are stress–strain curves, toughness and energy absorption.

Bonatti et al. [143] studied octet truss lattices with filled and hollow trusses by FE and experiments, obtaining Young’s modulus and stress–strain curves. Moussa et al. [221] analysed defects in octet truss structures. Their results demonstrated the roles of cell orientation and manufacturing defects in inducing anisotropy and affecting mechanical properties. Variations of the octet truss lattices are the double- and triple-octet truss structures [224].

While many more examples can be found in the literature, most of them lack generality, as they compare specific geometries through experiments and finite element models. The analysis of an experiment by a model offers some additional information in the sense that stress gradients and concentrations can be identified, but it does not provide a means to extend the results beyond the single geometry studied. The limited generality is also highlighted by the fact that most models of periodic 3D solids focus on uniaxial compression. This may be useful for energy-absorbing applications, but it is insufficient for the structural design of lightweight components.

Several important studies on elastic anisotropy are available [139,140,225], noting that a cubic lattice has only three independent elastic constants. Therefore, only three different, well-chosen stress states must be modelled. In mechanical design, the highest allowable stress in the material must be known in addition to its stiffness. For compressible media, defining a failure surface or yield surface requires the exploration of the full 6-dimensional stress or strain space. As a reference, a widely cited micromechanical model for calculating the yield locus of incompressible polycrystalline solids (in the 5-dimensional Π-plane) requires the evaluation of 402 strain modes [226]. Reducing the complexity of the yield locus to the Hill 1948 description [227] (modified to account for the compressibility of the medium) significantly reduces the number of simulations required [122].

The works cited above rely on extensive FEMs in which the entire unit cell, or a volume consisting of multiple unit cells, is meshed with 3D elements. One approach to reduce computational cost is to recognise that the trusses can be modelled as 1-dimensional beam elements, which is inherent to the original GA model. The elastic anisotropy of the tetrakaidecahedral cell has been calculated analytically [40]; the yield criterion of the octet truss lattice has been modelled under the assumption of pin joints, limiting the analysis to uniaxial stress in the trusses [228]. Storm et al. [229] presented an analytical solution for the yield surface of a Kelvin cell constituted by Euler–Bernoulli beams. Goda and Ganghoffer presented a model of a hexagonal cell using Timoshenko beams [230]. Chen and Tan used the same approach to predict the yielding of octet truss lattices [231].

While most of these approaches may have been promising at the time of their presentation, Zhong et al. [41] have clearly shown that the use of Euler–Bernoulli beams is only relevant for foams that have a density so low as to preclude their use as structural materials. This means that the models discussed above are oversimplifications. It also follows that the usefulness of GA to estimate the strength and stiffness of structural CS is limited. Instead, the authors present finite element models based on Timoshenko beam elements, which allows for a considerable reduction in computational cost compared to a detailed 3D mesh of all the trusses. They do not extend their work to yield surface calculations. Timoshenko beam elements have been used to predict the full yield surface of an open Kelvin-cell aluminium foam [232].

An entirely different approach is taken by Giorgio et al. [233] and Moschini et al. [234] who describe the energy-density function of pantograph and zigzagged parallelogram structures using gradient elasticity. This allows them to describe the stress–strain behaviour of the material as a homogeneous representative volume element directly in a user subroutine for existing finite element codes. This reduces the complexity of the mesh for numerical simulations of mechanical tests by several orders of magnitude.

### 5.2. Sheet-Based Unit Cells and TPMS

Classical sheet-based unit cells include the closed-wall Kelvin cell [40,114,127,129,130,142] and hollow-sphere packings [143,235]. More recently, TPMS have attracted attention for their regular and smooth curved surface geometries without discontinuities [46] and are attractive for their mechanical performance [236]. TPMS topologies have potential advantages over truss-based structures for bone fixation [150]. TPMS-based foams can be used as lightweight cellular materials for acoustic absorption, battery electrodes and catalyst support [237]. Some of them are similar to biological systems, such as butterfly wings and beetle shells [147]. Abueidda et al. studied the thermal, mechanical, and electrical properties of different TPMS-based structures and showed that they outperform other reported geometries [238].

**Figure 8:**
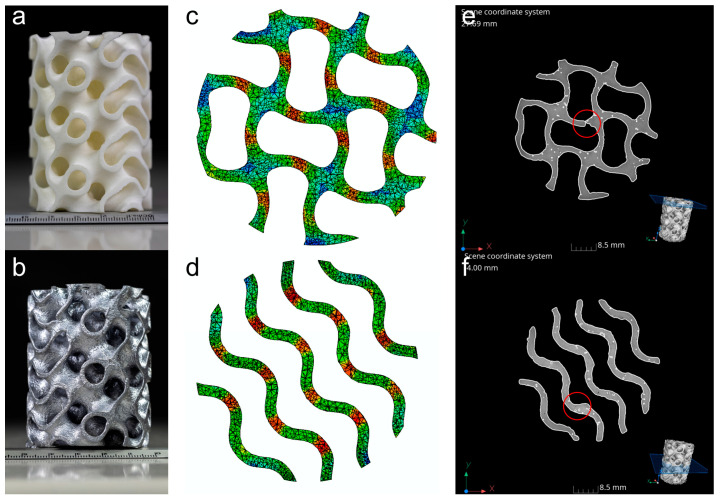
(**a**) Nylon model of a sheet-based gyroid. (**b**) Same geometry obtained by replication casting with a eutectic Al-Si alloy. (**c**,**d**) Finite element results (von Mises stress under compression) of sections through the idealised geometry. (**e**,**f**) X-ray tomography of the same sections, showing irregularities in wall thickness (red circles) and microporosity, which add randomness to the structure and reduce its resistance.

Unlike truss-based materials, the mechanical response of TPMS and closed-wall cellular solids can only be described accurately by FEM. Gyroid cells (Figure 8) are used to idealise foam materials. The specific energy absorption of gyroids is almost three times that of BCC structures with similar porosity [239]. Lee et al. [236] modelled a Schwarz primitive cell and presented a systematic investigation of several sections through the 6-dimensional stress space. They used a compressibility-corrected version of the Hill 1948 yield surface, which provided a good fit to their modelling results.

Abueidda et al. [240] generated Schwarz primitive, IWP, and Neovius PA2200 polymeric structures and tested them in tension and compression, finding reasonable agreement with FEMs. Chen et al. [241] studied four TPMS materials using an FFT-based homogenisation method, obtaining results with shorter calculation times than FEM. They analysed hybrid materials combining two structures, i.e., Schwarz Primitive + Neovius, and compared the effective Young’s, shear, and bulk moduli. Baghous et al. [242] obtained sections through the yield surface in the 6-dimensional stress space via FEM analysis of five different TPMS cells under axisymmetric, biaxial, double shear, shear, and hydrostatic stresses.

Skeletal TPMS are a variation of sheet TPMS, where the volume separated by the TPMS is filled to create a solid structure. The skeletal gyroid and skeletal diamond structures have been studied for their manufacturability and mechanical properties [243]. Skeletal TPMS lattices exhibit a more uniform, smooth transition stress distribution than truss-based structures, making them good candidates for tissue engineering [244]. Skeletal TPMS have proven to be suitable scaffolds in bone repair. Compression and bending tests demonstrated that skeletal gyroid scaffolds were flexible enough to promote bone healing, contrary to sheet gyroid scaffolds [245].

A purely experimental study varying the sheet thickness of a gyroid structure subject to fatigue was presented by Xu et al. [246]. The optimisation of TPMS-derived structures for mechanical and thermal properties using genetic algorithms was explored by Kaleghi et al. [247] and Ji et al. [248]. By extending the mathematical description of the TPMS toward level sets of the same [249], an additional design parameter can be introduced for the optimisation of the structure [141,247,250].

### 5.3. Random Structures

Stochastic foams are useful materials due to their low relative density, high specific strength and effective energy absorption [165]. The use of Voronoi tessellations is the most common approach to modelling random structures; the main difference between studies lies in the definition of the tessellation seeding points. A purely Poisson–Voronoi approach in 3D was pioneered by Gan et al. [251], who used Timoshenko beam elements in the FEM analysis of random truss-based structures. The use of specialised graphical software allows the introduction of struts with variable diameters, which require 3D elements [252]. Timoshenko beam elements have been used to model an open cell tetrakaidecahedron lattice with superimposed irregularity [253]. Chen et al. modified the irregularity of their cubic mesh of seeding points to adjust to the experimental pore size of their material [254]. The perturbation of a regular grid of seeding points was used to model closed-cell aluminium foams with good precision [255].

Hooshmand-Ahoor et al. [256] used the random sequential adsorption algorithm (RSA) [257] to create a volume with embedded spherical voids in an incompressible, nonlinear elastic matrix. By gradually growing the voids, a Voronoi tessellation is approximated. At intermediate densities, this method produces structures similar to those observed in Figure 2a. The study showed that the corresponding structures were both stronger and stiffer than spinodoid [258] and gyroid-based geometries. By far, the most popular method to construct random foam geometries for FEM is the Laguerre tessellation [111,112,113,259,260,261,262,263,264].

Multiple studies of random aluminium foams characterise uniaxial loading [252,254,262,263], biaxial loading [255], and fracture [253,264]. Vengatachalam et al. [255] obtained yield points by FE and biaxial compression/tensile testing. Triaxial tests and models for aluminium foams [265,266,267,268,269] demonstrate tension–compression asymmetry; the yield surfaces deviate significantly from Von Mises’ isotropic criterion for incompressible materials.

One of the most comprehensive studies on the multiaxial yielding behaviour of closed-cell foams was presented by Jung et al. [249], who investigated the distribution of the material along the trusses and joints of an open-cell aluminium foam and found a significant effect on the size and shape of the yield surface. Chen et al. [254] simulated foam models with varying sizes and levels of irregularity and obtained stress–strain curves for static and dynamic compression. Han et al. [253] simulated fracture in foam models with different defects, i.e., missing walls, cell shape irregularity, and nonuniform distribution of cell wall thickness.

Models for polymer foams based on X-ray tomography [270,271] have been used to simulate compression tests in polymethacrylimide [272] and polyvinyl chloride [273]. Shafiq et al. [274] used a custom-built triaxial testing device to cover the entire spectrum of multiaxial loads from hydrostatic compression to hydrostatic tension in PVC foams. The most impressive body of work in this field is the modelling of the yield surface of trabecular bone. Based on a statistically representative sample of X-ray tomography scans, realistic voxel-based FEMs have been constructed and tested under multiaxial loading conditions. Proposed yield criteria include the superellipsoid [275], modified superellipsoid [276], Tsai-Wu [277], and modified quadratic [278,279] yield surfaces, all of which are capable of capturing tension-compression anisotropy. Similarly to the role of the face sheet in sandwich materials, cortical bone can significantly alter the effective properties of trabecular bone [280].

### 5.4. Gradient Structures

There are three main strategies to introduce a gradient in cellular solids: (a) by varying the truss diameter [281] or wall thickness, (b) by scaling the unit cell [282], and (c) by varying the type of unit cell [283]. Gradients are generally defined by an increase in thickness from the centre to the outer layers of a component, as the latter are subject to higher loads under bending and torsional loading. The performance of graded lattice structures varies according to the type and magnitude of the gradients. Graded structures have been used in orthopaedic implants due to their porous characteristics, mimicking natural bone [284].

Connecting the unit cells of graded structures smoothly is a challenge. One solution is to thicken the joints to enhance connection stability [285], but this method cannot guarantee smooth transitions. Another solution is to adopt an intermediate transition body to connect different layers within the structure for a smooth transition [286], but this approach increases the weight and alters the structure’s topology. The problem is absent in a lattice structure generated by varying the unit-cell rod diameter [281,285,287,288]. Tamijani et al. proposed a Fourier series-based approach for designing smoothly varying gradient structures [289].

Analysis of the mechanical response of graded structures is generally limited to experimentation, but recently, numerical simulation has been adopted to verify their design. Wu et al. [290] proposed a bi-graded 2.5D honeycomb with in-plane (variable wall thickness along the edge direction) and out-of-plane (variable wall thickness along the height direction) gradients, and simulated compression tests using FEM. It was found that the thickness gradient of honeycombs played a significant role in the specific energy absorption (SEA) and peak crushing force (PCF) of the structure. Bai et al. [291] compared a size gradient BCC structure to a rod-diameter gradient BCC structure manufactured in a PA2200 polyamide and simulated by FEM. They conclude that a size-graded structure exhibits better strength at small strains and superior energy absorption ability at large strains. Yue et al. [122] studied gradient octet truss structures and obtained yield surfaces through theoretical, experimental, and FEM investigations.

Voronoi tessellation is an important method for simulating random gradient lattice structures. Using a controlled heterogeneous distribution of seeding points, anisotropic foams with a functional gradient can be designed. Wang et al. [292] used a Voronoi tessellation seeded on a perturbed regular array of points with a pre-existing point density gradient. By varying porosity and irregularity, they could, to a certain degree, modify the compressive stiffness and strength of their simulated foams independently. Duan et al. [293] tested graded foams with twelve different gradient distributions by additive manufacturing and FE. Graded foams with similar density exhibited a similar behaviour in the elastic and densification stages. However, graded foams showed an increasing strength during the plateau stage rather than a constant strength during compression at intermediate strains.

### 5.5. Use of Artificial Intelligence for the Prediction of the Mechanical Properties of CS

The use of artificial intelligence (AI) to predict material properties has grown over the last five years. AI is defined as the field of computer science that enables computers to mimic human intelligence processes, such as learning, self-correction, and reasoning [294]. It encompasses different methods, including machine learning (ML), traditional rule-based programming, and deep learning (DL). Traditional rule-based programming involves defining a set of rules and conditions for AI systems to follow.

ML involves training an AI model on large datasets to learn patterns and make predictions autonomously. In relation to property prediction, it is used to overcome the curse of dimensionality when the number of parameters involved in the design becomes too high to rely on classical response surface methodology. Instead, systematic or random sampling of the parameter space is performed, and the structures defined by the chosen parameters are tested experimentally or by numerical modelling [295,296].

ML can be classified by its method of learning: supervised learning can be used for regression and classification and is most used for property prediction; unsupervised learning recognises patterns in data without being specifically trained on labelled data. Support Vector Regression (SVR), Decision Tree (DT), and Artificial Neural Network (ANN) are some ML models. DL is a subset of ML that uses more than two hidden layers in an ANN to process complex data and is increasingly used to predict the mechanical properties of CS. Generative adversarial networks (GANs), convolutional neural networks (CNNs), and recurrent neural networks (RNNs) are among the most widely used DL models [297,298,299].

In AI-based prediction, data are divided into three datasets: a training dataset, a validation dataset and a testing dataset. The training dataset is used to train the AI model, enabling it to learn patterns and relationships within the dataset. The validation dataset is used to detect overfitting, which occurs when a model focuses too much on the training data and fails to predict new data. This can be prevented by controlling the learning process. The testing data are an independent collection used to assess the predictive performance on different examples.

Most of the papers that use AI to predict material properties focus on compression tests. Hooshmand et al. [300] simulated 360 different polyethene-walled structures, including TPMS, honeycombs and lattices, using 5 geometric parameters to train five different ML algorithms (linear regression (LR), polynomial regression (PR), random forest (RF), DT, and ANN) to predict the stiffness of polymer foams. The ANN model demonstrated the best prediction performance. Ma et al. [301] collected 57 samples from previous compression tests for 11 TPMS and lattice structures and used SVR to effectively predict the elastic modulus and yield strength. Reddy et al. [302] tested 35 different TPMS, honeycombs, and lattice structures of different sizes under different conditions (classified as built, heat-treated, and stress-relieved). They used a random forest (RF) algorithm to predict the ultimate strength, hardness, lattice volume, and surface roughness of the structures. Lattice volume was identified as the most influential factor in predicting displacement in compression tests. Thirupathi et al. [303,304] predicted the yield strength of finite element lattice structures (diamond and octet truss) and TPMS (Schwarz primitive, Schwarz diamond and gyroid) by RF, LR, SVR and gradient Boost Regression (GBR) methods. The predictions were compared with experimental results, where GBR showed the best predictions.

Multiple papers have tested the capabilities of AI models by predicting the compressive strength of foams. Aluminium [305,306,307,308,309] and polymer foams [310,311,312] were the most common, and the data were collected from experiments and simulations (geometries obtained by computed tomography or generated using Voronoi tessellation). Other tests used are bending [313], tension [314,315], relaxation [316], and impact testing [317]. Sahib and Kovács [313] studied the bending behaviour of composite sandwich structures with different honeycomb cores using modelling data as an input. The data were used to train an ANN. The model was validated using FEM and experiments, with good agreement. Mahapatra et al. [315] trained an RF model on experimental data to predict the yield strength, tensile strength and tensile failure strength of TPMS with different densities. The tensile failure strain predictions were affected by process parameters during manufacturing conditions.

Yüksel et al. [317] employed a DL-based generative adversarial network (GAN) to design lattice structures with tailored mechanical properties. The model was trained on 1827 alternative lattice designs generated with parametric design tools, each with specified constraints. Six unit cells generated by the GAN were chosen and compared with four classic unit cells (square, diamond, diamond squared, and body-centred). The mechanical performance of the additive manufactured unit cells was evaluated through FE, compression, and impact testing, with mechanical strength improving by up to 108%. A fundamental aspect of their research was to use classical optimisation techniques to ensure that only high-performance structures were used for model training, thereby generating only high-performance results from the GAN.

Mualla et al. [318] developed a methodology to generate mechanical metamaterials while ensuring the manufacturability of the predicted structures by creating a dataset spanning a broad range of mechanical properties by applying rotations to cubic structures and by training a physics-guided neural network (PSNN) to generate a unit cell with a desired anisotropic stiffness. The study of Matsuoka et al. [319] focuses on the filtering process for topology optimisation of 2D periodic lattice structures. Filtering techniques were used to consider the connectivity of lattice members. In a purely analytical study, they concluded that the models achieve higher accuracy when filtered data are used as input. Challapalli et al. [320] studied the design of multifunctional cellular structures based on different ML (RF, SVR and GAN), continuously producing novel and optimised structures. The efficacy of the cellular structures was validated through FE and compression tests. The predicted structure exhibited up to 120% higher performance than the octet unit cell. This work demonstrated significant potential to continuously optimise the design to meet specific mechanical property requirements.

Zheng et al. [321] developed a controllable design for auxetic metamaterials using the DL model. A conditional GAN model was trained to generate geometric patterns from input labels for mechanical properties. FE simulations and compression tests validated the design. The conclusions highlighted the potential to accelerate the design and development of 3D metamaterials. Hassanin et al. [322] tested different DL models (shallow neural network (SNN), deep neural network (DNN), and deep learning neural network (DLNN)) to achieve a diamond-shaped lattice structure. The DLNN achieves the best results. ML models were constructed by varying strut length, diameter, and orientation angle as input data and Young’s modulus and specific strength as output data. Yu et al. [323] designed heterogeneous lattice structures with customizable mechanical properties by an ANN model with five hidden layers. Compression FE simulations were conducted to confirm the mechanical properties of the proposed structure. Heterogeneous lattice structures exhibited greater energy absorption than octet-truss and rhombic dodecahedron lattices.

## 6. Summary Tables

### 6.1. Classification by Geometry

In the following, the topics analysed in the text will be summarised as tables. Notice that none of the tables that follow provide a list of references, because the goal of this work is not to make a quantitative meta-analysis of the literature. A more exhaustive search will always find additional references to fill the gaps in the table where the present review failed to cite the corresponding studies. The focus of the paper is on modelling strategies, not to provide a list of all possible CS that have been produced in recent history.

Most proposals for classifying cellular materials employ a tree-like structure, in which each class of materials is subdivided into smaller classes as additional characteristics are added [48,101,102]. It follows from this section that all material classes can be produced using multiple production techniques, covering a broad range of applications and geometries and defining a finite set of discrete points on a mathematical lattice rather than a tree. It shall be reminded that a lattice is the equivalent of a vector space, with the basis vectors multiplied by integer numbers $z_{i}\in Z$, instead of real $r_{i}\in R$. This is illustrated in Table 2.

Classification of materials as metals, ceramics and polymers can be based on rigorous definitions in physical chemistry, with a few exceptions. Classification of composite materials is much more complex; overlaps and exceptions can be found in most proposed schemes. This will also be the case for CS. Whether the subdivision open/close cell and truss/sheet-based should be treated as independent criteria can be discussed.

For purely 2D solids, which only exist as models, plane stress conditions correspond to very thin trusses, while plane strain corresponds to very long sheets. For 2.5D and 3D solids, truss-based structures can never be closed cell. Any 2.5D honeycombs without face sheets are open-cell sheet-based, while the face sheet, in general, converts the structure to closed cell. Sheet-based TPMS and some sintered sphere structures can be classified as sheet-based open-cell structures; hence, the distinction makes sense.

An advantage of Table 2 is that it proposes a convenient set of abbreviations. For example, a 2.5D, gradient open-cell truss-based solid can be written as 2.5D-GOT-Y, where Y can be M, C, P, Cmp, or Bio for metals, ceramics, composites, or biomaterials, respectively. More generally, the code becomes XD-UVW-Y, where X can be 1.5, 2, 2.5, 3, 3.5 or 4, U takes the values of P, R or G (periodic, random, gradient), B can be O or C (open or closed), and W becomes T or S (truss or sheet). This scheme defines 360 lattice points in a five-dimensional lattice, with not all of them occupied.

### 6.2. Classification of Randomness

Another concept that merits classification, or even definition, is randomness. If one or more stochastic variables are involved in the description of the structure of the CS, the structure is random. To properly describe randomness, the probability density function of each stochastic variable involved must be defined, for example, uniform, exponential, or Gaussian, with a given mean and standard deviation. For the purpose of the present paper, random processes are defined by the stochastic nature of the spatial coordinates defining the geometry of the structure, or by the stochastic nature of the absence or presence of given structural elements at determined positions within the structure.

An attempt for classification is given in Table 3. A distinction is made between cell geometry, which is defined as the description of the polygons (2D, 2.5D) and polyhedra (3D) defining the structure on the one hand, and structural elements on the other, the latter being the edges and corners (2D, 2.5D) or vertices, edges, and faces (3D) of the cells.

### 6.3. Summary of Manufacturing Techniques

While some manufacturing processes are adequate to produce a broad range of CS, most of them are very specific. In Table 4, the manufacturing techniques cited in this paper are summarised, using a primary subdivision of traditional manufacturing and additive manufacturing. Biological materials are not considered, as they grow naturally. Additive manufacturing is highly flexible in terms of the geometry, so a distinction between periodic, random and gradient is less important here, but many techniques are restricted to open cells, as remaining powder, reagents and support structures may have to be removed.

## 7. Discussion

Before starting a discussion of the state of the art in mechanical models for CS, it should be noted that the modelling of strain hardening [324,325,326], fracture [327], or fatigue [328,329,330] in conventional alloys remains a considerable challenge. It must therefore not be a surprise that there are no models available that provide a comprehensive description of the mechanical behaviour of cellular solids. Nonetheless, there are clear tendencies in the literature, both in the types of materials studied and in the modelling approach.

Two-dimensional models are used as idealised approximations for regular and random honeycombs, which are mainly employed in sandwich panels subjected to bending loads. Examples are packaging materials, aeroplane wings, ship hulls, and wind turbine blades. Two-dimensional models can only address in-plane loads and are therefore of little relevance for technological applications.

Even in the case of 2.5D models, in-plane loads are more widely studied than out-of-plane behaviour. Table 2 has many open entries, because modelling of 2.5D structures is often substituted by 2D models. However, a significant number of models describe bending [193], crushing [195,196] and the impact of sandwich panels [194]. General models based on the parameters that define the geometry and mechanical properties of constitutive materials are scarce; experiment-based failure maps have been explored [198]. Design engineers can rely on extensive technical specifications made available by commercial producers. The corresponding datasets were not reviewed in this work, as they are mostly based on extensive experimentation.

For random 3D foams, Voronoi tessellations with controlled statistical properties can predict the elastic behaviour and yield locus of a wide variety of materials using FEM [113,251,252,253,254,255,256,259,260,261]. For open-cell foams with beams of constant section, the computational efficiency of this approach can be increased by using Timoshenko beam elements [251,253]. The main challenge appears to be establishing a relationship between the distribution of nucleation points for the tessellation and the microstructural parameters of the foam [254]. This aspect is addressed in bone modelling by directly using FEM meshes derived from micro-CT scans of a statistically representative set of trabecular bone samples at a considerable computational cost [276,277]. It has also been applied to polymer foams [272,273,274].

For periodic 3D cellular solids, a clear evolution is observed in both the geometries studied and the modelling approaches. Early studies on wire-mesh lattices, investment-cast structures, and additive manufactured components used truss-based materials, with a focus on the GA model and its extensions. These models are not predictive, as they require parameter calibration. TPMS and related surfaces have gained attention over the last decade. They can be described by a single or a few parameters, which allow for the systematic variation and optimisation of their properties [247,248,250]. The effect of joint geometry on truss-based solids has been a topic of discussion [168,169,170,252]. Recent results indicate that the use of smoothly varying surfaces is advantageous due to reduced stress concentrations [21,42,141,143,180,236,250].

Truss-based materials can, in theory, be analysed by the methods of structural mechanics, as demonstrated by the GA model. Surprisingly, only a few studies have explored this possibility [40,63,124,206,230,231,253]. Several examples exist as pure 2D idealisations, and others consider pin-jointed trusses, which are not generally found in cellular solids. Euler–Bernoulli beams have been generally used; however, it has been demonstrated that trusses in structural materials do not comply with the corresponding slenderness requirement. FEM studies using Timoshenko beam elements are promising in this field [41,63,230]. The opportunities in this field are largely unexplored.

Most models rely on brute-force 3D FEM, which is computationally much more expensive but requires less intellectual input on behalf of the modeller. For smooth honeycombs, TPMS-related materials, and trusses with varying diameters, FEM is the only viable option. Producing high-quality finite element meshes that capture the geometric complexity of structures remains a challenge, and the number of elements tends to be very high. Many studies model the entire sample used in experiments, while others reduce the computational effort by analysing a single unit cell. In both cases, the exact definition of the boundary conditions is critical to obtain correct results [44,133,141].

Many models have a limited scope, focusing on a single loading condition, typically compression. For energy-absorbing materials under impact and crushing conditions, this may be justified. For the structural design of lightweight components, the elastic stiffness matrix must be determined together with a yield surface, or more generally, a failure surface. Stiffness and strength will be important in most designs, but resilience ($\frac{\sigma_{yC}^{2}}{2E_{C}\;}$) may be important on some occasions and favours materials such as the gyroid and similar TPMS. Whether isotropy is important will depend on the specific operation conditions.

In summary, among the many modelling studies reviewed in this paper, very few provide specific guidance on selecting a particular geometry for the structural design of lightweight components. There are several exceptions to this observation, such as Karri and Kambagowni [180] and Zhong et al. [41], and, more specifically, the extensive tables of results in the supplementary information of their papers. It has been shown that smooth honeycombs are superior to polygonal honeycombs because of the absence of stress concentrations [174], as was the case when comparing truss-based solids to TPMS-based geometries. Interesting studies on the parametric optimisation of such materials are available [93,141,241,246,247,248,250] and may serve as a basis for optimising a broader range of cellular solids.

The use of AI does not render these observations obsolete, as mechanical models are required to train the AI models. A correct definition of the design parameters for the geometries to be studied is crucial in this field. Studies focusing on a narrow set of geometries or material classes [300,317] seem more effective than very broad studies [302]. Previous optimisation of the structures used by classical parametric design enhances the convergence of the AI model toward optimal solutions [316,320]. Alternatively, AM may be used to produce structures that can be tested experimentally. This may be an essential step in the design of optimised products, as numerical models rarely include the omnipresent production defects inherent to AM and replication casting techniques (Figure 8).

When considering the design of lightweight structures under any loading conditions, the experimental approach may become excessively expensive. In contrast, an FEM mesh, once optimised, can accommodate a wide range of stress states. In this sense, it must be noted that the use of AI remains limited in scope. At present, most studies focus on establishing the feasibility of the approach, with the best AI model to use and training methods for a limited set of loading conditions. Models, whether classical or AI-based, that enable the design engineer to find the optimal CS for a given application are not yet available.

Furthermore, a significant disparity remains between idealised numerical predictions and the mechanical response of physical specimens, primarily due to the omission of manufacturing-induced defects in current modelling frameworks. Experimental evidence demonstrates that stochastic irregularities, such as microporosity and variations in wall thickness, are inherent in both replication casting and additive manufacturing processes. These imperfections are not merely aesthetic; for instance, the presence of missing or broken cell walls can reduce the macroscopic yield strength by more than 60%, with stretch-dominated architectures such as the Kagome lattice exhibiting particular sensitivity to such distributed defects.

Consequently, for cellular solids to be reliably integrated into safety-critical structural applications, there is an urgent requirement to transcend the ‘brute-force’ FEM of perfect geometries. The use of energy-density descriptions of the structure to define the equivalent homogeneous medium is an interesting approach here [234,235]. Future research should prioritise the development of stochastic sensitivity analyses and high-fidelity models that incorporate realistic defect distributions, thereby transforming models from tools for experimental confirmation into robust instruments for predictive engineering design.

## 8. Conclusions

The Gibson–Ashby model has been fundamental in establishing the study of the mechanical properties of cellular solids; however, it is not a valid tool for developing new materials with enhanced mechanical properties or for structural design of lightweight components. More refined beam-based models following a structural mechanics approach have been insufficiently explored and may provide an efficient means for the parametric study of the corresponding cellular solids.

The gradual shift from truss-based materials to smooth-surface geometries makes the use of FEMs unavoidable. Most of these models have a narrow focus, which prevents them from extending their results to the field of materials selection and structural design. Models should be used to predict properties rather than confirm experimental results, i.e., a large set of numerical simulations should be used to define a narrow set of experiments under critical conditions for model validation.

The introduction of AI in the field has shifted the focus to the parametric description of the geometry of cellular solids. Classical parametric optimisation has been insufficiently studied but is now being incorporated to generate training data for AI models. At the moment of writing, AI has not yet moved to the level of user applications for materials selection and design. Methods that allow for finding an optimal structure for a given application under a broad range of loading conditions are not yet available. This leads to the widespread use of well-known but often suboptimal configurations, such as hexagonal honeycombs or octet truss lattices.

## Figures and Tables

**Table 2:** Classification of cellular solids. The letters indicate the research methodology used in the papers cited in the text (B: Euler–Bernoulli beams, T: Timoshenko beams, S: shells, F: finite element model, M: machine learning, A: advanced curved beam models [124,203,204,205]). Empty cells mean that no examples of the corresponding CS were cited in this work; greyed-out cells correspond to classes of CS that make no physical sense or that do not exist.

Geometry				Materials				
				Metals	Ceramics	Polymers	Compos.	Biomat.
2D	Random	Open cell	Truss					
			Sheet					
		Closed cell	Truss	F	F	F		B, F
			Sheet					
	Periodic	Open cell	Truss					
			Sheet					
		Closed cell	Truss	B, F, M, S	B, F, M	B, F, M	F	
			Sheet					
	Gradient	Open cell	Truss					
			Sheet					
		Closed cell	Truss					
			Sheet					
2.5D	Random	Open cell	Truss					
			Sheet					
		Closed cell	Truss					
			Sheet					
	Periodic	Open cell	Truss	B, F, A	B	B, F	B	
			Sheet	B, F		B, F		
		Closed cell	Truss					
			Sheet	F	F	F		
	Gradient	Open cell	Truss					
			Sheet					
		Closed cell	Truss					
			Sheet					
3D	Random	Open cell	Truss	B, T, F, M		B, T, F, M		T, F
			Sheet	F, M		F, M		
		Closed cell	Truss					
			Sheet	F		F		
	Periodic	Open cell	Truss	B, T, F, M	B	B, F, M		
			Sheet	F, M		F, M		
		Closed cell	Truss					
			Sheet	F		F		
	Gradient	Open cell	Truss	F, M		F, M		F
			Sheet	F		F		
		Closed cell	Truss					
			Sheet	F		F		

**Table 3:** Classification of randomness in CS.

Cell geometry	Tesselation	Poisson	Homogeneous
			Heterogeneous
		Centroidal	Homogeneous
			Heterogeneous
		Laguerre	Homogeneous
			Heterogeneous
	Random perturbation of regular nodal positions		
Component geometry	Random removal of trusses/shells		
	Random variation of component geometry (dimensions)		
	Production defects		

**Table 4:** Summary of manufacturing techniques for CS. (SLS: selective laser sintering, LPBF: laser powder bed fusion, EPBF: electron beam powder bed fusion, SLA: stereolitography).

Manufacturing Process		Dimensions	Cell Arrangement	Open/Closed	Truss/Sheet	Material Class
Traditional	Wire Weaving	2.5D, 3D	P	O	T	M, P
	Knitting	2.5D, 3D	P, R	O	T	P
	Punching-corrugation	2.5D	P	O	S	M, P, Cmp
	Slotting/Interlocking	2.5D	P	O, C	S	M, P, Cmp
	Investment casting	2.5D, 3D	P, R, G	O, C	T, S	M
	Replication casting	2.5D, 3D	P, R, G	O, C	T, S	M, C
	Replication sintering	3D	R	O	T	M, C
	Sponge replication	2.5D, 3D	M, C	O	T, S	M, C
	Injection	2.5D, 3D	P	O	T, S	M, P
	Hot press moulding	2.5D	P	O	S	P, Cmp
	Vacuum-assisted moulding	2.5D	P	O	S	P, Cmp
	Self-foaming	3D	R	O, C	T, S	M, C, P
	Gas foaming	3D	R	O, C	T, S	M, C, P
	Blowing agent foaming	3D	R	O;C	T, S	M, C, P
	Powder metallurgy	3D	R	O	T	M, C
	Hollow-sphere sintering	3D	P, R	O, C	S	M, C
	Combustion synthesis	3D	R, G	O, C	S	C
Additive	SLS	3D	P, G, R	O	T, S	P
	LPBF	3D	P, G, R	O	T, S	M
	EPBF	3D	P, G, R	O	T, S	M
	Binder jetting	3D	P, G, R	O	T, S	M, C
	Direct ink writing	3D	P, G, R	O, C	T, S	C, P
	Fused filament extrusion	2.5D, 3D	P, G, R	O, C	T, S	P, Cmp
	Photopolymerisation SLA	3D	P, G, R	O	T, S	C, P
	Digital Light processing	3D	P, G, R	O	T, S	M, C, P
	Material Jetting	3D	P, G, R	O	T, S	M, C, P, Cmp
	Directed energy deposition	3D	P, G, R	O, C	T, S	M

## Data Availability

No additional data are available beyond those published in the reviewed papers.

## Abbreviations

The following abbreviations are used in this manuscript:

FEM	Finite element models
3D-CS	3D cellular solids
GA	Gibson and Ashby
LPBF	Laser powder bed fusion
ϕ	Porosity
$P_{C}$	Property of cellular solid
$P_{S}$	Property of constituent material
$\rho_{C}$	Density of cellular solid
$\rho_{S}$	Density of constituent material
$\rho_{R}$	Relative density
Γ	Geometry of the beams or walls
CS	Cellular solids
TPMS	Triply periodic minimal surfaces
HEM	Homogeneous equivalent material
WBK	Wire-woven bulk kagome
RSA	Random sequential adsorption algorithm
PVC	Polyvinyl chloride
SEA	Specific energy absorption
PCF	Peak crushing force
